# Storage stability of soy protein isolate powders containing soluble protein aggregates formed at varying pH

**DOI:** 10.1002/fsn3.1759

**Published:** 2020-08-11

**Authors:** Fengxian Guo, Luan Lin, Zhiyong He, Zong‐Ping Zheng

**Affiliations:** ^1^ Fujian Province Key Laboratory for Development of Bioactive Material from Marine Alge College of Oceanology and Food Science Quanzhou Normal University Quanzhou Fujian China; ^2^ State Key Laboratory of Food Science and Technology Jiangnan University Wuxi Jiangsu China

**Keywords:** net protein charge, pH treatments, protein aggregation, Soy protein isolate powder, storage stability

## Abstract

Soy protein is wildly used in food industry due to its high nutritional value and good functionalities. However, the poor storage stability of commercial soy protein products has puzzled both the producers and the users for a long time. The current study assessed the changes in protein solubility, aggregation, oxidation, and conformation of soy protein isolate (SPI) with various soluble aggregates formed at different pH values (pH 5–8) during storage. During storage, SPI samples showed a reduced protein solubility (*p* < .05), an increased protein oxidation (*p* < .05), and an attenuated conformational enthalpy (∆H). SPI with a higher pH produced more disulfide‐mediated aggregates at the expense of sulfhydryl groups and experienced greater losses of protein tertiary structure and a faster reduction in solubility. Yet, all samples nearly shared similar rising trend during 8‐week storage, which indicated the production of protein carbonyls was insensitive to pH. Soluble aggregates present in fresh SPI samples appeared to induce instability of SPI during storage. These findings suggested SPI prepared at pH 6 was in favor of its storage stability, and soluble aggregates presented in fresh samples should be paid more attention for further study of storage stability kinetics.

## Graphical abstract

SPI powder with higher pH (before spray drying) exhibited a lower storage stability. Higher pH produced more disulfide‐mediated aggregates. More soluble aggregates in fresh SPI appeared to induce faster loss of protein solubility.

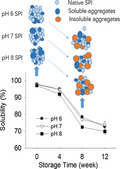

## 1 INTRODUCTION

Soy protein is widely used in food formulations, nutraceutical products, and beverages as an inexpensive functional and health‐promoting ingredient (Wang, Liu, Ma, & Zhao, 2019). However, the protein solubility of soy protein often decreases during storage (Martins & Netto, 2006; Pinto, Lajolo, & Genovese, 2005). For example, soy protein isolate (SPI) solubility decreases by about 63% after 1‐year storage at 42°C (Pinto et al., 2005). This insolubility of soy protein seriously affects its commercial applications because solubility plays an important role in the gelling and emulsifying properties of this functional ingredient (Hua, Cui, Wang, Mine, & Poysa, 2005). Therefore, to meet the high demand of soy protein for food, nutraceutical, and beverage applications, several studies have been conducted to investigate the factors that might impact the structural and functional properties and the mechanism involving in storage instability of soy protein in the solid state.

The relative humidity (RH) and temperature are two main environmental conditions that affect the storage stability of soy protein. High RH and high temperature have been found to accelerate the losses of SPI solubility during storage (Liu et al., 2008; Martins & Netto, 2006; Qinchun, Klaassen Kamdar, & Labuza, 2016; Shih, Hwang, & Chou, 2016). Additionally, protein oxidation might also associate with the decline of soy protein stability (Hellwig, 2019). Boatright and Hettiarachchy (1995, 2006) reported that the protein oxidation occurred along with the decrease of SPI solubility during storage, and the addition of antioxidants during processing significantly improved protein solubility.

The mechanism of protein insolubility in solid state is related to protein denaturation, aggregation, and oxidation (Alam, Siddiqi, Chturvedi, & Khan, 2017; Chang & Pikal, 2009; Guo et al., 2015). Non‐native protein aggregation occurs in five steps: (a) partial unfolding of protein molecules, (b) reversible protein self‐associations, (c) protein rearrangements, (d) protein aggregate growth, and (e) protein aggregate complexes. Protein aggregation results in the formation of precipitates or gels (Andrews & Roberts, 2007; Brummitt et al., 2011; Svilenov & Winter, 2019). Therefore, protein unfolding contributes to protein aggregation.

Protein unfolding can be induced by physical and chemical methods (Wang, 2005). Theoretically, raising or lowering the pH from the isoelectric point (p*I*) will increase protein net charges (Malhotra & Coupland, 2004), which could cause protein unfolding through disrupting intramolecular ionic bonds (Bera & Nandi, 2014; de Oliveira et al., 2018; Jiang, Chen, & Xiong, 2009). During SPI processing, pH treatments are applied, for example, extraction at pH 8.0, precipitation at approximately pH 4.5, and neutralization at pH 7.0 (Jiang et al., 2009). Furthermore, decreasing the pH in the direction of p*I* could result in a change in aggregation kinetics from second to first‐order in protein concentration (Olsen, Andersen, Randolph, Carpenter, & Westh, 2009; Schein, 1990). Additionally, pH plays an important role in a protein's oxidative susceptibility (Li, Schöneich, Wilson, & Borchardt, 1993). For example, adjusting the pH to alkaline levels promotes oxidation as evidenced by the conversion of sulfhydryls to disulfide bonds, sulfonic, and sulfenic acids (Barelli et al., 2008). The increased attraction of prooxidative metal ions (Me^2+^) by negatively charged amino acid side chain groups at the alkaline condition would promote protein oxidation as well. Hence, pH treatments may contribute to SPI storage instability as a result of protein aggregation and oxidation. However, the effect of pH treatments on SPI storage stability has not been reported.

In the present study, we investigated the effect of pH on protein aggregation, oxidation, and solubility of SPI. Before lyophilization, SPI samples were adjusted to different pH values (pH 5, 6, 7, and 8) to encompass a broad range of pH conditions under which SPI is used as a key component in the formulation of dry mixes and blends of pH‐specific beverages, soups, gravies, or other prepared foods and additives. The SPI samples were then analyzed for protein solubility, aggregation, degree of oxidation, and conformation. The objective of this study was to understand the effects of pH on protein aggregation and solubility so as to provide useful information for the establishment of appropriate pH for dry SPI ingredient preparation and subsequent long‐term storage.

## 2 MATERIALS AND METHODS

### 2.1 Sample preparation

SPI was prepared from defatted soybean flakes donated by Yihai Jiali Co. (Qinhuangdao, China) following the procedure of Jiang et al. (2009). The resulting SPI was spray dried. The dry SPI powder was dispersed in deionized water (8%, w/v) and magnetically stirred for 1 hr at room temperature (22°C). The protein content of the SPI solution at pH 7 was 94.6% (on a dry basis) based on the micro‐Kjeldahl method (N × 6.25). The SPI solution was adjusted to pH 5, 6, 7, or 8 using 1 M HCl or 1 M NaOH before lyophilization. Freeze‐dried samples were ground in a mortar and pestle. Water activities of all these samples, which were checked with a dew point water activity meter (Aqualab 4TEV, Decagon Devices, Inc. Pullman, WA, USA) at 25°C, were around 0.42–0.45. The samples were vacuum‐packaged in composite film (PET/NY/AL/PE) bags (O_2_ permeability 0.024 ml/m^2^.24 hr at 0.1 mPa 25°C; water permeability 0.006 g/m^2^.24 hr at 90% RH 40°C) and stored at 37°C in an incubator for 4, 8, and 12 weeks. Even after 12 weeks, the physical condition of the packages (i.e., tightness, shrinkage, and wrinkles) showed no sign of change, indicating that the hypobaric condition was well maintained through the storage period.

### 2.2 Protein solubility

SPI samples were dispersed in deionized water at a 2% (w/v) concentration. The solutions were adjusted to pH 7.0 with 1 M HCl or 1 M NaOH and magnetically stirred for 1 hr at room temperature, then were centrifuged at 10,000g for 15 min at 20°C. Protein concentration in the supernatant was determined by the micro‐Kjeldahl method. Protein solubility was calculated as the percentage of protein concentration in the supernatant relative to the protein concentration in the SPI solution.

### 2.3 Zeta potential

SPI samples were dispersed in deionized water at a 2% (w/v) concentration and magnetically stirred for 1 hr at room temperature. The solution was centrifuged at 10,000g for 15 min, and the resulting supernatant was diluted to 5 mg/ml with deionized water. Zeta potential of SPI was measured by Laser Doppler Electrophoresis using a Nano Zetasizer dynamic light scattering instrument (Nano‐ZS, Malvern Instruments Ltd., Worcestershire, UK). Electrophoretic mobility was measured in a well‐defined electric field at 150 V and 250 Hz. The instrument was calibrated with a standard carboxyl‐modified polystyrene latex solution with a zeta potential of –55 mV obtained from Malvern Instruments Ltd. (Worcestershire, UK). Zeta potential was calculated using the Helmholtz–Smoluchowski equation.

### 2.4 Molecular weight (MW) distribution

Gel permeation chromatography (GPC) was used to determine the MW distribution of the SPI samples. SPI solution (2%, w/v) was centrifuged at 10,000g for 15 min. The supernatant was diluted to 10 mg/ml with distilled water and filtered through a 0.45‐μm filter. Protein separation was performed with 10 μL of the diluted SPI supernatant in an HPLC instrument (LC‐20A; Shimadzu Co., Tokyo, Japan) equipped with a PROTEIN KW‐804 column (8.0 × 300 mm; Shodex, Tokyo, Japan) and a UV‐detector (SPD‐M20A, Shimadzu Co., Tokyo, Japan) (280 nm). The column has an exclusion size limit of 1,000 kDa for globulins. Phosphate buffer (50 mM, pH 7.0) containing 0.3 M NaCl was used as eluent; the flow rate was set to 1.0 ml/min and the eluate was monitored at 280 nm. All samples were measured in duplicate; representative results were selected for analysis and discussion. An LCsolution Workstation (Shimadzu Co., Tokyo, Japan) was used to analyze the area under the peaks. To estimate the MW of protein particles and aggregates in SPI samples, a standard curve was constructed using the following MW markers: thyroglobulin (660 kDa), amylase (200 kDa), alcohol dehydrogenase (150 kDa), albumin (66 kDa), carbonic anhydrase (29 kDa), and cytochrome C (12 kDa).

### 2.5 Electrophoresis

SDS–PAGE was performed following the procedure described by Liu and Xiong (2000). SDS–PAGE was operated using a vertical slab gel of 1.0 mm thickness with a 4% stacking gel and a 12% resolving gel in a Bio‐Rad mini‐protein electrophoresis system (Bio‐Rad Laboratories, Hercules, CA, USA). An SPI solution (2%, w/v) was centrifuged at 10,000g for 15 min. The supernatant was diluted to 2 mg/ml with distilled water; 30 μL of the diluted supernatant was mixed with an equal volume of sample buffer with and without 5% (v/v) *β*‐mercaptoethanol (*β*ME). All mixtures were boiled for 3 min before SDS–PAGE.

### 2.6 Protein sulfhydryl (SH) group content

Protein SH group content was determined according to the method of Hoshi and Yamauchi (1983). An SPI solution (2%, w/v) was stirred for 1 hr and centrifuged at 10,000g for 15 min. Supernatant aliquots (1 ml) were added to 2 ml of phosphate buffer (0.1 M, pH 8.0) containing 0.05 ml of 0.01 M 5,5‐dithiobis‐2‐nitrobenzoic acid (DTNB) and 6 M guanidine hydrochloride. Absorbance at 412 nm was measured after allowing the solutions to stand for 20 min at 30°C. SH content was calculated using the molar absorption coefficient of 13,600 M^‐1^ cm^‐1^. Results were expressed as µmol SH/g protein.

### 2.7 Protein carbonyl group content

Protein carbonyl group content in SPI was assayed by the method of Levine et al. (1990). An SPI solution (2%, w/v) was stirred for 1 hr and centrifuged at 10,000 g for 15 min. The resulting supernatant (500 μL) was incubated with 500 μL of 2,4‐dinotrophenynylhydrazine reagent for 1 hr at 30°C and mixed in a vortex every 10–15 min. Approximately 500 μL of trichloroacetic acid (TCA) was added and the solution was allowed to stand for 10 min at room temperature. The solution was then centrifuged at 11,000 g for 3 min, and the supernatant was discarded. The resulting pellets were washed three times with 1 ml of ethyl acetate‐ethanol (1:1, v/v) and allowed to stand for 10 min at room temperature each time before centrifugation. To dissolve the precipitated protein, 0.6 ml of 6 M guanidine was added and the solution was allowed to stand for 1h at 37°C. Insoluble materials were removed by centrifugation at 11,000 g for 3 min. A blank was prepared with 2 M HCl instead of 2,4‐dinotrophynylhydrazine. Spectra were measured at approximate 350–390 nm and read against the blank. The protein carbonyl group content was calculated from the maximum absorbance using a molar absorption coefficient of 22,000 M^‐1^ cm^‐1^. Results were expressed as μmol carbonyl/g protein.

### 2.8 Calorimetric Measurement

Protein denaturation of the SPI samples was analyzed by DSC (Q 2000 DSC, TA Instruments, New Castle, DE, USA). SPI powders were dispersed in distilled water (20%, w/v). Solutions (14–18 mg) were sealed in aluminum pans and an empty pan was used as reference. The pans were heated from 25 to 120°C at a rate of 5°C/min. Each sample was measured in triplicate, and an average value was reported.

### 2.9 Statistical analyses

Data were obtained from three independent replicates. Data were analyzed by one‐way ANOVA using the SPSS program (SPSS Statistical Software, Inc., Chicago, IL, USA). Statistical significance was set at *p* < .05; differences between means were identified by the least significant difference (LSD) test.

## 3 RESULTS AND DISCUSSION

### 3.1 Protein solubility

**FIGURE 1:**
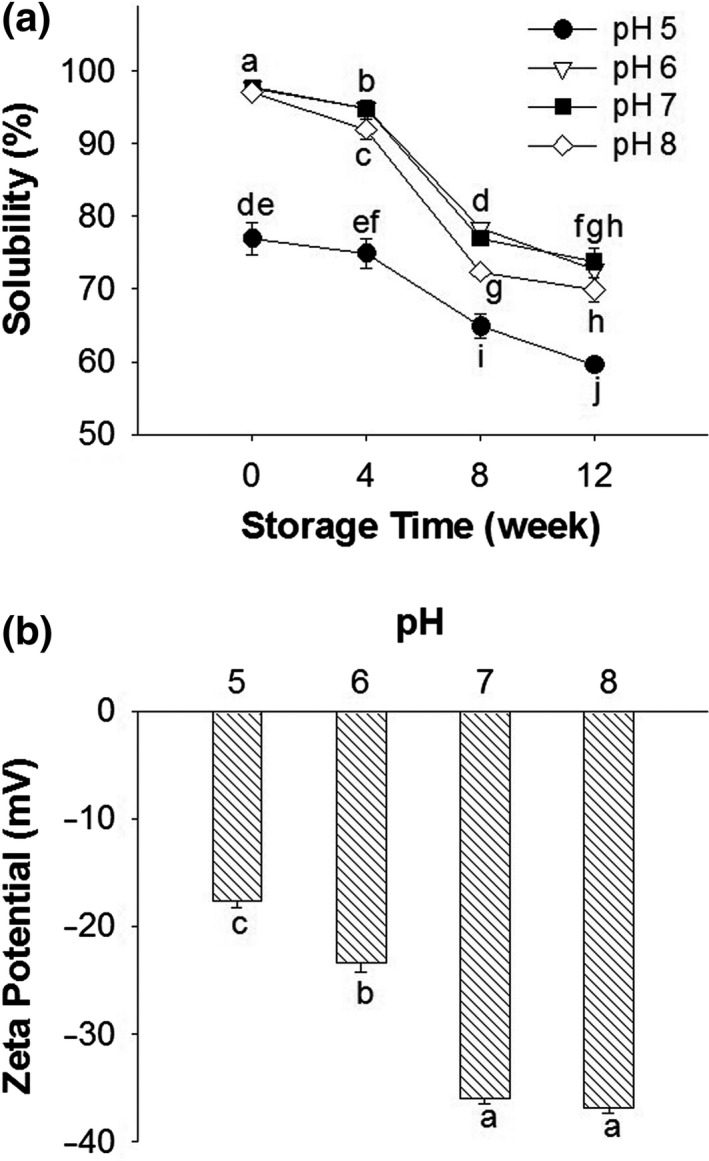
(a) Protein solubility of SPI powders at different pH values (pH 5, 6, 7, or 8) and storage periods (0, 4, 8, or 12 weeks). (b) Zeta potential of SPI powders at different pH values (pH 5, 6, 7, or 8). Means with three replications (*n* = 3) in all the samples with different letters differ significantly (*p* < .05)

To explain the solubility change and the difference between pH samples, Zeta‐potential of the initial samples was measured. As shown in Figure 1b, the magnitude of the zeta potential increased with increasing the treatment pH value, suggesting that a relative higher pH value could induce a higher electrical potential of protein molecules in SPI. As shown in Figure 1(a), the different decreasing degree of protein solubility among the samples was thought to be essentially due to their different content of net charges. Among samples, pH 5 SPI showed the lowest protein solubility throughout storage since the pH value is close to the isoelectric point of globulins in SPI (~pH 4.5) where protein solubility is always sensitive to small changes in the environment pH (Jiang et al., 2009). On the other hand, other pH samples were almost completely soluble at the beginning, and some chemical effects appeared to involve in them. At increasing pH from 6 to 8, there would be an increased thiol reactivity, which could promote the formation of aggregates through the disulfide linkages. Moreover, at increasing pH, there may be an increased hydrophobicity due to the disruption of intramolecular ionic bonds, leading to a greater susceptibility to hydrophobic aggregation (Jiang et al., 2009).

### 3.2 Molecular weight (MW) distribution

**FIGURE 2:**
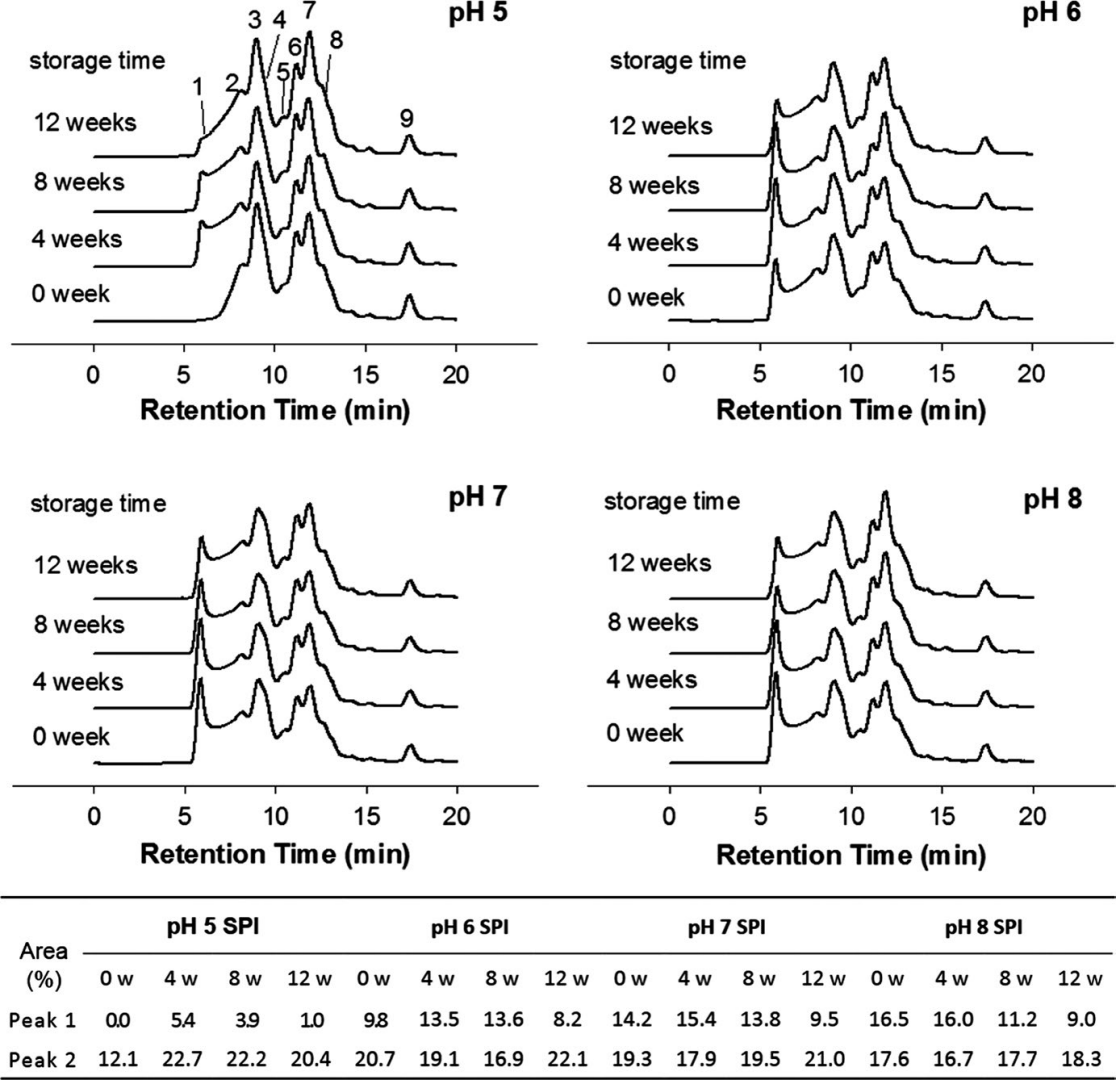
Molecular weight (MW) distribution of an SPI solution (5 mg/ml) at different pH values (pH 5, 6, 7, or 8) and storage periods (0, 4, 8, or 12 weeks). Peak 1: ≥10,000 kDa; peak 2:670–10,000 kDa; peak 3:350 kDa; peak 4:170 kDa; peak 5:70 kDa; peak 6:30 kDa; peak 7:14 kDa; peak 8 and 9: <10 kDa. Insert table: the area percentage of peak 1 and 2 in different samples

While the above solubility measurement identified the magnitude of insoluble protein aggregate formation, as suggested by Guo et al. (2012), the production of “transient” polymers that are hydrodynamically stable and separable by gel filtration can reveal the tendency of permanent insoluble aggregate formation during SPI storage. Therefore, gel permeation chromatography (GPC) was employed to characterize soluble protein particles that coexisted in SPI suspensions. As displayed in Figure 2, native SPI yielded nine major protein peaks. Peaks 1 and 2 were categorized as large‐size (≥1,000 kDa) and mid‐size (660–1000 kDa) soluble aggregates. Based on the MW proximity, peaks 3 and 4 were designated as 11S and 7S globins at 350 kDa and 170 kDa, respectively; peak 5 was assigned to the AB complex of 11S and also to the *α*, *α´*, *β* subunits of 7S (70 kDa); peaks 6 and 7 at 30 kDa and14 kDa, respectively, were most likely A and B polypeptides; peaks 8 and 9 were small MW fractions (<10 kDa) yet uncharacterized.

In fresh samples, higher pH SPI contained higher amounts of soluble aggregates (peaks 1 and 2) (Figure 2 insert table). In pH 5 SPI, peak 1 was barely visible, whereas in pH 8 SPI, peak 1 amounted to 16.5% of the total protein. To a lesser extent, samples at pH 6 and 7 also contained large‐size soluble aggregates before storage (9.8% and 14.2%, respectively). As aforementioned, charge repulsions at high pH values could result in protein unfolding (Kristinsson & Hultin, 2003), which would lead to the exposure of hydrophobic areas in both glycinin (11S) and *β*‐conglycinin (7S) molecules of SPI (Jiang, Xiong, & Chen, 2011) and promote protein aggregation in later storage duration. These findings indicated a direct relationship between pH value and the quantity of these aggregates when results from all the pH samples were considered.

The influence of pH on the protein aggregation demonstrated a dynamic process. As shown in Figure 2, the pH 5 samples were more stable than the other samples during storage. Yet, the amount of large‐size soluble aggregates (peak 1), which were virtually absent in nonstored pH 5 samples, progressed to 5.4% by week 4, then, surprisingly, declined to 1.0% by the end of storage (week 12). In the pH 6 and 7 samples, the changes in the content of these large‐size aggregates during storage followed a rather similar trend to that of the pH 5 samples where the content of the aggregates rose to 13.5 and 15.4% during the first 4 weeks before dropping to 8.2 and 9.5% by week 12, respectively. In contrast, in the pH 8 samples, the proportion of the large‐size aggregates consistently declined throughout the 12‐week storage. The decrease in the soluble aggregate content was accompanied by the formation of insoluble fractions (Figure 1a), suggesting major shifts in chemical bonds and other intermolecular forces leading to the transformation of aggregates from a hydrodynamic state to a permanent and hydrophobic state.

The production of insoluble aggregates was ostensibly related to the content of soluble aggregates in the samples at the beginning of storage (week 0). There have been suggestions that freshly prepared SPI can readily form soluble aggregates, but these aggregates have the tendency to transform into insoluble polymers (Chi, Kendrick, Carpenter, & Randolph, 2005; Roberts, 2003, 2007). Samples at pH 5, 6, and 7 shared the same aggregate formation‐transformation pattern, namely, during the first 4 weeks, the formation of soluble aggregates was significant, while protein solubility showed no appreciable change. From week 4 to week 8, there was a major reduction in the amount of soluble aggregates with a concomitant decrease in protein solubility due to the conceivable formation of insoluble aggregates. Because the initial content of soluble aggregates was relative high in the pH 8 samples, the aggregate transformation process was significant during storage, leading to a faster protein solubility reduction in the pH 8 samples than in the other samples.

### 3.3 SDS‐PAGE

**FIGURE 3:**
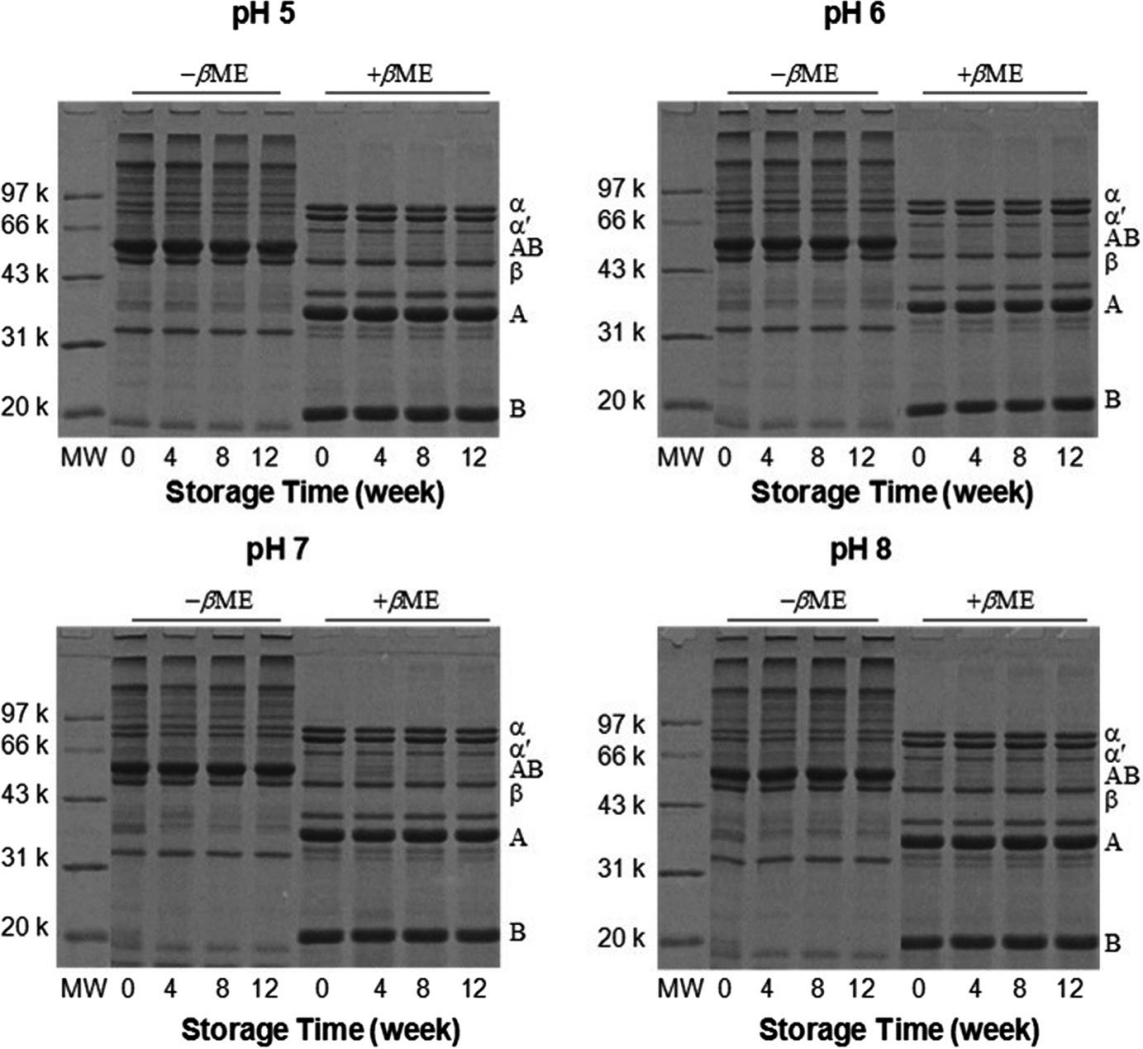
SDS‐PAGE of SPI powder at different pH values (pH 5, 6, 7, or 8) and storage periods (0, 4, 8, or 12 weeks). SDS‐PAGE samples had 5% *β*‐mercaptoethanol (+*β*‐ME) or no *β*‐mercaptoethanol (‐*β*‐ME). MW: molecular weight marker (Da). SPI constituents: *αˊ*, *α*, and *β* for conglycinin; A, acidic subunit of glycinin; B, basic subunit of glycinin

Electrophoresis of the soluble SPI fraction with or without *β*ME was performed to determine the role of disulfide bonds in the formation of protein aggregates in SPI powders of different pH values. Under nonreducing condition (i.e., –*β*ME), extremely large aggregates appeared on the top of the stacking as well as the resolving gels (Figure 3). The aggregates had a higher proportion in higher pH samples, for example, the percentage of aggregates in the –*β*ME samples was 34.8% in the pH 8 sample, more than 27.3% in the pH 5 sample (calculated by density percentage). This result was in accordance with the GPC results (Figure 3). When *β*ME was applied, all samples exhibited similar electrophoretic patterns. The large polymers were almost completely dissociated into A and B subunits of 11S and, to a lesser extent, α, α′ subunits of 7S as well. This result is very reasonable since the content of SH and/or SS groups is higher in 11S than that in 7S (Nielsen, 1985). Therefore, S‐S cross‐linking of 11S and, less appreciably, 7S, was mainly responsible for the nonhydrophobic aggregates formed in SPI during storage. Furthermore, since the insoluble aggregates were developed from soluble aggregates (Barelli et al., 2008) and there was no obvious new band in the reduced samples, it is possible that the presence of hydrophobic aggregations and the formation of disulfide linkages were the two main interactions that participated in protein insolubility. Petruccelli and Añón (1995) reported that the typical thermal soluble aggregate in SPI, β‐7S/B‐11S polymer, was stabilized by hydrophobic interactions and later by SS bonds.

### 3.4 Protein oxidation

**FIGURE 4:**
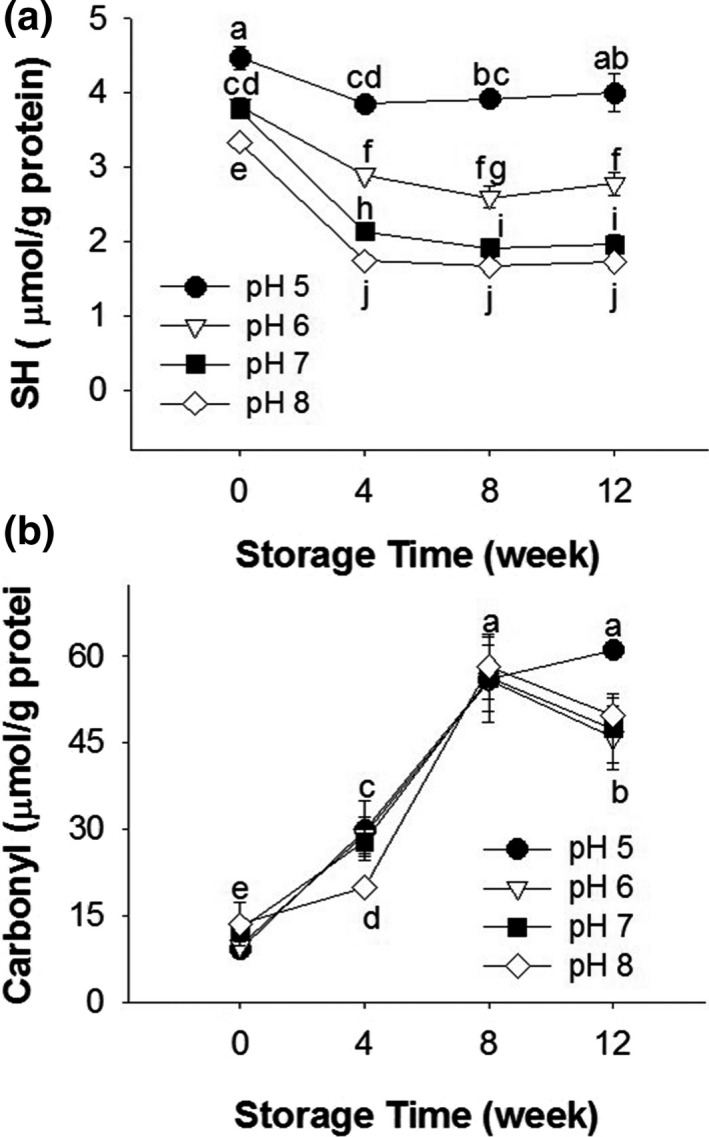
Free sulfhydryl group (SH) (a) and carbonyl group (b) content of SPI samples at different pH values (pH 5, 6, 7, or 8) and storage periods (0, 4, 8, or 12 weeks). Means with three replications (*n* = 3) in all the samples with different letters differ significantly (*p* < .05)

Free SH group content in SPI was affected by sample pH values and the storage period. As shown in Figure 4a, in freshly prepared samples, the highest SH content was present in the pH 5 sample, and the lowest was obtained in the pH 8 sample. All the samples shared similar tendency of SH content change during storage, in which the SH content markedly declined during the first 4 weeks, then changed insignificantly later on. The magnitude of SH disappearance was greater in the pH 7 and 8 samples than that in the pH 5 and 6 samples. These results were consistent with that obtained above, in which pH 5 sample showed highest stability. The decline in SH groups of SPI during storage was also reported by Duque‐Estrada, Kyriakopoulou, de Groot, van der Goot, and Berton‐Carabin (2020), which was mainly due to the oxidation of thiol groups, which could have produced disulfide linkages (through SH and SS exchange), sulfenic acid, and sulfinic acid (Barelli et al., 2008). As revealed by the results of SDS–PAGE, the loss of SH groups in SPI samples was probably to form new intermolecular SS bond, thus to produce more SS‐mediated soluble aggregates.

The DNPH assay provided additional information on protein oxidation, that is, the production of protein carbonyl groups. As shown in Figure 4b, the content of protein carbonyl groups increased sixfold after an 8‐week storage period; a similar tendency was observed in other samples. This finding suggests that although all samples were vacuum‐packaged, the oxidation of protein in SPI powders occurred during storage. It was reported that even 1% oxygen might be sufficient to produce complete oxidation of protein (Chang & Pikal, 2009). Furthermore, protein oxidized by free radicals and metal‐catalyzed reactions could result in aggregation (Huang, Hua, & Qiu, 2006; Stadtman, 1993). Studies have reported that the oxidation of muscle protein could reduce its solubility (Smith, 1987). In this study, the increase of carbonyl was consistent with the decrease of protein solubility during 8‐week storage period, thus the oxidation in SPI powder might limitedly contribute to protein aggregation. However, this carbonyl‐generating oxidation was not directly related to the decline in protein solubility since oxidation was not affected by pH variation in the SPIs.

### 3.5 Conformational characteristics

**FIGURE 5:**
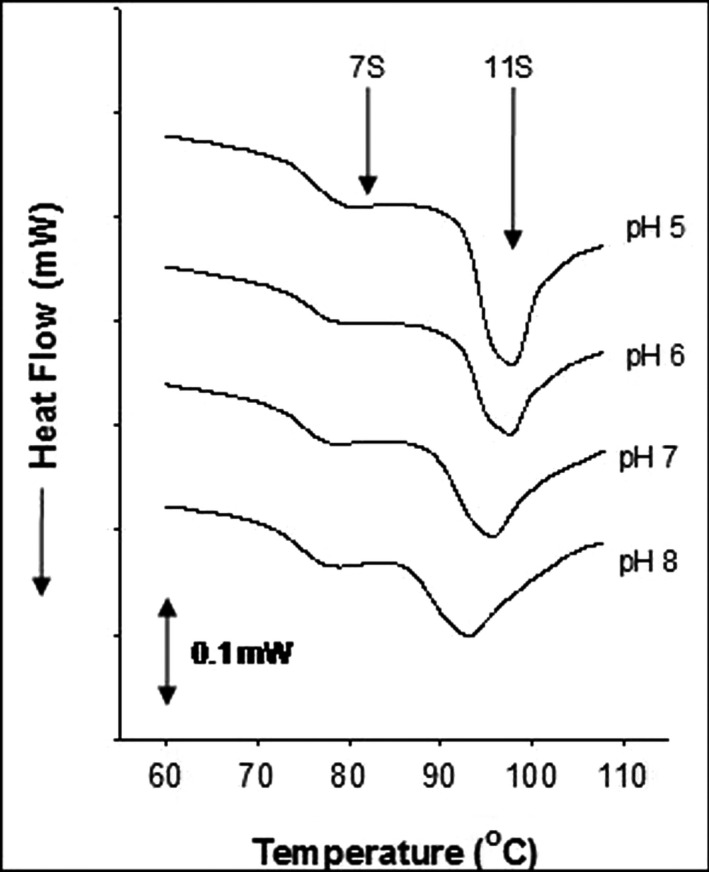
Representative DSC curves of SPI powder at different pH values (pH 5, 6, 7, or 8) at the beginning of storage

Because protein aggregation is intimately related to the conformational stability, that is, tendency to unfold, DSC was applied to elucidate the structural compactness of SPI powders stored for different time periods. As shown in Figure 5, all samples exhibited two endothermic peaks: 7S at approximately 77.4–79.2°C and 11S at approximately 93.2–97.8°C. Protein (7S and 11S) denaturation enthalpies (∆H, J/g) and temperatures (T_d_, °C) of SPI with different pH values are shown in Table 1. The ∆H value of 7S and 11S decreased after 12 weeks of storage in all the samples. Also, the highest denaturation degree (calculated as the decline proportion of ∆H) of both 7S and 11S was obtained in pH 8 samples (at 12 weeks), and the lowest one was found in pH 5 and 6 samples. The loss of ∆H usually suggests a change in the protein conformation (e.g., unfolding) and a reduction in stability (Jiang et al., 2009; Kristinsson & Hultin, 2003). Increasing the pH values from 5 to 8 resulted in a drop of 11S T_d_, suggesting that SPI with higher pH value had lower thermal stability. The same phenomena were also observed by Hermansson (1978). The T_d_ reduction is common in globular proteins because protein molecules tend to be most stable against denaturation when they have low net protein charges (e.g., when the pH value is close to the p*I* value) (Hermansson, 1978). The results of ∆H and T_d_ were in accordance to that of GPC (Figure 2), SH content (Figure 4a), and protein solubility (Figure 1a). SPI with pH 5 showed highest stability as revealed in DSC analysis, corresponding to the lowest degree of protein aggregation, the least magnitude of reduction in SH groups and protein solubility; contrary results were observed in pH 8 sample. These results suggested that pH of SPI before freeze‐drying significantly affected thermal stability; high pH of SPI induced protein unfolding, which resulted in the production of large‐size aggregates and storage instability of freeze‐dried SPI powder.

**Table 1:** Denaturation enthalpy (∆H) and denaturation temperature (T_d_) of SPI powders at different pH values (pH 5, 6, 7, or 8) and storage periods (0, 4, 8, or 12 weeks)

Sample	storage time (week)	7S fraction		11S fraction	
		∆H (J/g)	T_d_ (°C)	∆H (J/g)	T_d_ (°C)
pH 5	0	1.8 ± 0.4^a^	79.2 ± 0.1^a^	11.5 ± 0.2^a^	97.8 ± 0.1^abc^
	4	1.3 ± 0.0^cde^	79.3 ± 0.2^a^	9.5 ± 1.3^ab^	98.0 ± 0.0^ab^
	8	1.4 ± 0.2^abcd^	78.5 ± 0.6^abcd^	9.7 ± 1.0^ab^	97.5 ± 0.3^abc^
	12	1.4 ± 0.1^abcde^	78.5 ± 0.2^abcd^	9.3 ± 1.0^bc^	97.5 ± 0.1^abc^
pH 6	0	1.4 ± 0.1^bcde^	78.3 ± 0.0^abcde^	8.8 ± 0.6^bcd^	97.6 ± 0.1^abc^
	4	1.4 ± 0.1^bcde^	78.5 ± 0.1^abcde^	9.7 ± 0.3^ab^	97.8 ± 0.1^abc^
	8	1.0 ± 0.2^e^	77.5 ± 0.1^cde^	8.1 ± 0.7^bcde^	97.2 ± 0.3^abcd^
	12	1.3 ± 0.2^cde^	78.4 ± 0.8^abcde^	7.4 ± 0.1^cdef^	98.2 ± 0.9^a^
pH 7	0	1.8 ± 0.1^ab^	77.5 ± 0.4^de^	9.2 ± 0.5^bc^	95.3 ± 0.7^ef^
	4	1.3 ± 0.2^cde^	78.0 ± 0.4^bced^	7.9 ± 1.2^bcde^	96.3 ± 0.0^cde^
	8	1.3 ± 0.1^cde^	77.6 ± 1.1^cde^	8.1 ± 1.1^bcde^	96.2 ± 1.0^cde^
	12	1.3 ± 0.2^cde^	78.0 ± 0.2^bced^	6.3 ± 0.1^ef^	95.7 ± 1.0^de^
pH 8	0	1.6 ± 0.1^abc^	77.4 ± 0.0^de^ 78.7 ± 0.3^abc^ 79.1 ± 1.4^ab^ 77.3 ± 0.3^e^	9.7 ± 1.1^ab^	93.2 ± 0.1^g^
	4	1.7 ± 0.2^abc^		8.9 ± 0.3^bcd^	95.7 ± 0.2^de^
	8	1.4 ± 0.3^cde^		7.0 ± 1.5^def^	96.5 ± 2.2^bcde^
	12	1.1 ± 0.1^de^		5.6 ± 1.7^f^	93.8 ± 0.6^fg^

Note:

Means with three replications (*n* = 3) with sample quality parameter with different letters differ significantly (*p* < .05). The data are presented as means ± *SD*. Means within a row with different letters are significantly different by the least significant difference (LSD) test.

## 4 CONCLUSIONS

In conclusion, dry SPI powders at different pH values were remarkably different in their storage stability as evidenced by the aggregation structural stability, solubility, and oxidation tests. In general, SPI stability was increased by the reduction of pH (from 8 to 5). The conversion of soluble aggregates formed in the early stage of storage to insoluble protein polymers was mainly due to the disulfide linkages and hydrophobic interaction. These findings indicated that monitoring the pH value to 6 was beneficial for storage stability of SPI. Also, for freshly prepared SPI, besides protein solubility, soluble aggregates content might be another important index for predicting product storage stability. The kinetics of such reaction, the relationship between the level of residual air (oxygen) in the package, and the possible role of antioxidants need to be investigated in future studies.

## ETHICAL STATEMENTS

The authors declare no conflict of interest. This study has nothing to do with human and animal testing.
